# Outcomes of Irish graduate entry medical student engagement with self-directed learning of clinical skills

**DOI:** 10.1186/s12909-015-0301-x

**Published:** 2015-02-19

**Authors:** Deirdre McGrath, Louise Crowley, Sanath Rao, Margaret Toomey, Ailish Hannigan, Lisa Murphy, Colum P Dunne

**Affiliations:** Graduate Entry Medical School and Centre for Interventions in Infection, Inflammation & Immunity {4i}, University of Limerick, Limerick, Ireland

**Keywords:** Clinical skills, Self-directed learning, Graduate entry medicine, Student engagement

## Abstract

**Background:**

Existing literature is mixed as to whether self-directed learning (SDL) delivers improvements in knowledge, skills or attitudes of medical students compared with traditional learning methods. This study aimed to determine whether there is an association between engagement in SDL and student performance in clinical examinations, the factors that influence student engagement with SDL in clinical skills, and student perceptions of SDL.

**Methods:**

A retrospective analysis of electronic records of student bookings of SDL sessions from 2008 to 2010 was performed for students in the pre-clinical years of an Irish Graduate Entry Medical programme to assess their level of engagement with SDL. The extent to which this engagement influenced their performance in subsequent summative examinations was evaluated. A cross-sectional survey of students across the four years of the programme was also conducted to determine student perceptions of SDL and the factors that affect engagement.

**Results:**

The level of engagement with SDL decreased over time from 95% of first years in 2008 to 49% of first years in 2010. There was no significant difference between the median exam performance for any clinical skills tested by level of engagement (none, one or more sessions) except for basic life support in first year (p =0.024). The main reason for engaging with SDL was to practice a clinical skill prior to assessment and the majority of respondents agreed that SDL sessions had improved their performance of the specific clinical skills being practised.

**Conclusion:**

Students viewed SDL as an opportunity to practise skills prior to assessment but there were no significant differences in subsequent summative assessment by the level of engagement for most clinical skills.

## Background

The dynamic nature of medical knowledge and evidence-based practice, in addition to the need for maintaining professional competence, all reinforce the importance of preparing medical graduates to become life-long learners. Life-long learning is an on-going process, which leads to “systematic acquisition, renewal, upgrading and completion of knowledge, skills and attitudes”; its success depends on learners’ “increasing ability and motivation to engage in self-directed learning (SDL) activities” [1]. The concept of SDL was first outlined in the context of adult learning [2] and was defined as “ a process in which individuals take the initiative…in diagnosing their learning needs, formulating goals, identifying …resources for learning, choosing and implementing appropriate learning strategies, and evaluating learning outcomes”. In the context of medical education, there is inconsistency in how SDL is defined [3,4] but the key components of the original definition above should still be applied, in addition to the educator acting as a facilitator of learning.

Despite some initial uncertainty [4,5], SDL has been proposed as a means of emphasising the importance of life-long learning, particularly in the context of professional competence for medical professionals across many disciplines e.g. internal medicine [6], general practice [7], paediatrics [8], obstetrics and gynaecology [9]. Indeed, it has been widely adopted in the education of many healthcare professionals, although, arguably, most notably in the delivery of medical [10,11] and nursing [12,13] curricula.

In recent decades, medical schools are incorporating the educational strategy of Problem-based Learning (PBL) into their curricula to a lesser or greater extent. As it is a relatively innovative approach, much research has been done on PBL. However, due to lack of consistent definitions of PBL, and limited research of high quality, it is difficult to determine just how widespread its use is [14]. PBL facilitates knowledge acquisition by students with the “skills to deal with the information explosion through self-directed learning, information search and retrieval, critical appraisal, and self-assessment” [15-17]. The PBL approach aims to help students develop their problem solving skills, and in so doing, take responsibility for their own learning. In this regard, PBL shares many of the key components of SDL.

The increasing use of simulation in medical education [18-20] has resulted in the need for students to engage in deliberate practice of skills to achieve an acceptable level of competence. In medical schools, clinical skills training tends to occur in standardized, controlled and safe learning environments conducive to students “being shown what to do, practicing (where possible) on models, simulated patients or one another, performing (skills) under close supervision, obtaining feedback, and then practicing the skill with increasingly distant supervision until they are ‘licensed’ to perform the skill independently” [21]. However, recruitment of sufficient, relatively expensive, clinical teachers can be problematic and as students need to practise skills with increasingly distant supervision, SDL approaches to clinical skills training have become attractive and have expanded beyond the boundaries of effective pre-clinical teaching [22] into clinical and post graduate training. Indeed, they have been shown to be effective in some specific areas, including, for example, surgery [23], cardiopulmonary resuscitation [24] and anatomy [25,26].

Despite the demonstrated promise, a systematic review by Murad et al [27] concluded that SDL delivered only moderate improvement in the knowledge domain compared with traditional teaching methods and may be as effective in the skills and attitudes domains. Therefore, the potential for benefits in acquisition of skills and knowledge appears to vary depending on the skill, whether the learner is involved in identifying their learning resources, and the level of facilitation/supervision provided.

In this context, the objectives of this study were as follows: (i) to quantify the extent of student engagement with clinical skills SDL (as measured by student bookings of clinical skills labs); (ii) to determine whether there was an association between this engagement and student performance in subsequent summative clinical examinations; (iii) to determine the factors that affect this engagement, and (iv) to determine student perceptions of clinical skills SDL.

## Methods

This study was completed at an exclusively graduate-entry medical school (ULGEMS) established at the University of Limerick, Ireland in 2007 [28,29]. Previous reports have described the progress of this school and the academic development of its students [30,31]. At ULGEMS, clinical skills are taught twice weekly in formal teaching sessions and, usually, relate directly to the PBL case for that week, with students being trained in skills relevant to specific virtual clinical cases. Students also have the option of online booking of clinical skills laboratories and equipment to facilitate SDL. Students are informed that electronic records of these SDL bookings are retained.

Participants in this study were from diverse primary degree disciplines and had varying levels of post-graduate experience, ranging from registration in medical school in the semester immediately following undergraduate qualification to having a number of years working in their initial chosen field.

A retrospective analysis of the extent of student engagement with SDL was performed using the retained electronic records of SDL bookings for the academic years beginning September 2008-2010. Bookings were analysed with respect to students in the initial pre-clinical two years of the curriculum for: dates of the sessions and their proximity to clinical examinations, the skills/equipment requested for the session and the number of times each skill/equipment was requested. In addition to this, it was also determined whether a student booked the session personally or attended a session booked by a peer. The combined numbers of first and second year students in each year analysed are as follows: 97 (2008/09); 152 (2009/10); 188 (2010/11).

The relationship between subsequent performance in the end of year summative Objective Structured Clinical Examinations (OSCE) and attendance at booked physical examination and procedural skills SDL sessions was explored. The content of OSCE stations that assess procedural skills reflects the way that students are taught the skill and also the way in which the skills are practised in SDL.OSCE standards were set by applying the Angoff method to the checklist for each skill assessed [32]. This method involves subject-matter experts examining the content of each test question (item) and then predicting how many borderline candidates would answer the item correctly. The average of the experts’ predictions for a test question becomes its predicted difficulty. The sum of the predicted difficulty values for each item averaged across the experts and items on a test is the recommended Angoff cut-off score. As the maximum score achievable in a station can vary from 12 to 28, depending on the skill being assessed and the number of items on the checklist, results were therefore expressed as percentages for comparison.

Non-parametric tests (Mann-Whitney) were used to test for statistically significant differences between the median examination performances (expressed as percentages) across groups (no SDL sessions, one or more sessions). Where the number of sessions attended had a wide enough range to be treated as a numeric variable, Spearman’s correlation coefficient was used to measure the strength of the association between number of sessions attended and examination performance. A 5% level of significance was used for all tests and the analysis was performed using IBM SPSS Statistics for Windows version 20.0.

A cross-sectional survey of all medical students in the school (two pre-clinical years and two clinical years, n = 358 registered between 2008 and 2010) was carried out in 2012. Students were contacted by email and provided a link to the Survey Monkey^TM^ online study instrument and to a concise, unbiased explanation of the survey topic. Participation was voluntary and anonymous. The first question of the survey asked students to confirm that they consented to the study. This response and the subsequent completion of the questionnaire constituted participant consent. The study instrument determined the extent to which students felt they engaged with SDL; the group size engaging in SDL activities; the skills they practised most and least in SDL (fixed options); possible factors influencing their engagement with SDL, (including preparing for an imminent clinical examination), and finally, their perceptions of SDL. Agreement with statements given in the questionnaire was rated on a scale of 1 to 5 where 1 is disagree strongly and 5 is agree strongly. The survey also allowed students to add free text comments on the SDL aspect of the clinical skills programme.

Data were downloaded from Survey Monkey™ software to an electronic data file. Free text comments were analysed independently by two reviewers (LC, AH) to identify emergent themes. Researchers then met, discussed the themes emerging from the data, identified dominant themes and reached agreement around the clustering of themes into categories.

Conduction of the study and its design, taking into consideration a published survey on medical student responses to team-based learning and SDL [33], were approved by the Ethics Committee of the Faculty of Education and Health Sciences, University of Limerick, Ireland.

## Results

### Extent of student engagement with self-directed learning

**Figure 1:**
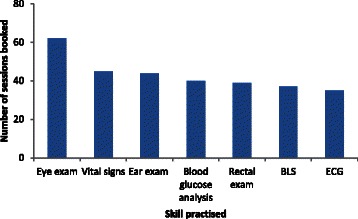
**Skills focused on by first year students (n = 99) during SDL sessions.** BLS: Basic Life Support ECG: Electrocardiograph.

**Figure 2:**
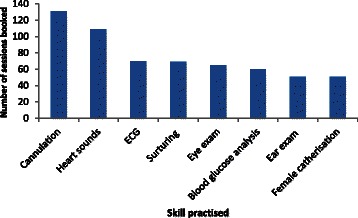
**Skills focused on by second year students (n = 89) during SDL sessions.** ECG: Electrocardiograph.

Taking those students participating in *any* clinical skills-related SDL, regardless of frequency and whether they personally made the booking of equipment, as reflective of engagement with SDL it was noted that engagement by students in the first year of their programme decreased on consecutive years (i.e; 95% 2008, 70% 2009, 49% 2010) (Table 1). There was a similar trend observed for students in second year over the same period (i.e; 97% 2008, 85% 2009, 78% 2010). However, while the numbers of students participating in SDL sessions decreased between 2008 and 2011, an increase in personal booking of SDL sessions between first and second year occurred in both of the cohorts assessed. It was also noted that students in first and second year engaged considerably more with SDL approximately two months prior to OSCE assessments (Table 1). This engagement was most pronounced in second year students (i.e; those being assessed for the third time). Skills/equipment most frequently booked in the academic year 2010/11 are shown in Figures 1 and 2 for first year and second year students respectively. It is noteworthy that the most frequently booked skills/equipment were common to students from both years of the course.

**Table 1:** **Student engagement with self-directed learning**

Academic year*	2008/09	2008/09*	2009/10*	2009/10**	2010/11**	2010/11
Year of curriculum	2	1	2	1	2	1
Total number of students in class	32	65	61	91	89	99
% engagement with SDL	97%	95%	85%	70%	78%	49%
% students personally booked SDL	47%	41%	43%	26%	35%	26%
% SDL bookings at 2 months before formative OSCE	n/a***	48%	n/a***	31%	n/a***	10%
% SDL bookings at 2 months before summative OSCE	82%	45%	68%	24%	65%	64%

*Same cohort of students progressing from Year 1 to Year 2 of the curriculum.

**Same cohort of students progressing from Year 1 to Year 2 of the curriculum.

***Formative OSCEs are not held in Year 2.

### Relationship between performance in examinations and clinical skills SDL engagement

Examination performance at the end of academic year 2010/11 was analysed. Students were divided into two groups based on the extent of their engagement with SDL (no engagement with SDL, participation in one or more SDL session) for the specific clinical skill subsequently tested in the OSCE. There was no statistically significant difference between the median examination performance by group for each clinical skill for first year students, with the exception of Basic Life Support (BLS) (p = 0.024) (Table 2). As the number of BLS sessions attended increased, the performance in the exam also tended to increase (Spearman’s correlation coefficient r_s_ = 0.3, p = 0.003). No other significant correlations were found. There was also no statistically significant difference between the median exam performance by group for each clinical skill for second year students (Table 3) though the results for phlebotomy tended to increase as the number of sessions attended increased (r_s_ = 0.30, p = 0.006). No other significant correlations were found. Interestingly, BLS results were lower (although not significantly) for second year students.

**Table 2:** **OSCE performance (percentages) at end of Academic Year 2010/11 for first year students (n = 99) by engagement with SDL**

Skill practised and tested	Number of SDL sessions per student median (min, max)	Exam performance (no SDL) median (min, max) n (% of total)	Exam performance (1 or more SDL sessions) median (min, max) n (% of total)
*Basic life support	0 (0, 5)	83 (38, 97) n = 81 (82%)	90 (76, 97) n = 18 (18%)
Eye examination	0 (0, 5)	85 (15, 100) n = 60 (61%)	85 (31, 100) n = 39 (39%)
Abdominal examination	0 (0, 1)	76 (36, 100) n = 97 (98%)	90 (88, 92) n = 2 (2%)
Female Ppelvic examination	0 (0, 3)	83 (65, 100) n = 79 (80%)	87 (61, 96) n = 20 (20%)
Blood pressure	0 (0, 5)	89 (22, 100) n = 67 (68%)	86 (33, 100) n = 32 (32%)
Cardiovascular examination	0 (0, 1)	75 (20, 95) n = 95 (96%)	75 (70, 85) n = 4 (4%)
Subcutaneous injection	0 (0, 3)	89 (43, 100) n = 73 (74%)	91 (68, 100) n = 26 (26%)
Peripheral nervous system (PNS) -sensory examination	0 (0, 1)	76 (47, 94) n = 93 (94%)	74 (65, 88) n = 6 (6%)
Cranial nerve examination	0 (0, 1)	75 (54, 96) n = 97 (98%)	84 (82, 86) n = 2 (2%)

*Mann-Whitney test comparing median exam performance across groups (p = 0.024).

**Table 3:** **OSCE performance (percentages) at end of academic year 2010/11 for second year students (n = 89) by engagement with SDL**

Skill practised and tested	Number of SDL sessions per student median (min, max)	Exam performance (no SDL) median (min, max) n (% of total)	Exam performance (1 or more SDL sessions) median (min, max) n (% of total)
Basic life support	0 (0, 2)	79 (46, 100) n = 61 (69%)	79 (50, 100) n = 28 (31%)
Hip examination	0 (0, 1)	62 (38, 97) n = 86 (97%)	72 (69, 76) n = 3 (3%)
Suturing	1 (0, 3)	63 (25, 100) n = 44 (49%)	70 (30, 100) n = 45 (51%)
Female pelvic examination	0 (0, 2)	78 (35, 100) n = 61 (69%)	83 (65, 96) n = 28 (31%)
Respiratory examination	0 (0, 1)	90 (67, 100) n = 83 (93%)	88 (76, 100) n = 6 (7%)
Cranial nerve examination	0 (0, 2)	75 (39, 100) n = 66 (74%)	75 (50, 89) n = 23 (26%)
Cardiovascular examination	0 (0, 1)	76 (62, 95) n = 84 (94%)	67 (62, 81) n = 5 (6%)
Phlebotomy	1 (0, 4)	79 (16, 95) n = 25 (28%)	89 (47, 100) n = 64 (72%)

### Results of survey of student perceptions of SDL

The overall response rate was 24% (n = 86) with similar rates of respondents across the first two preclinical and latter two clinical years of the curriculum. 62 (72.1%) of the 86 respondents had booked an SDL session themselves. Eighty-five of the 86 respondents had participated in an SDL session booked by other students. For SDL sessions booked by the respondents themselves, the number of students who participated in the session with them ranged from 0 to 6, with a median of 2 other students. For SDL sessions booked by other students, the number of students who participated in the session with them ranged from 2 to 15, with a median of 5 other students.

**Figure 3:**
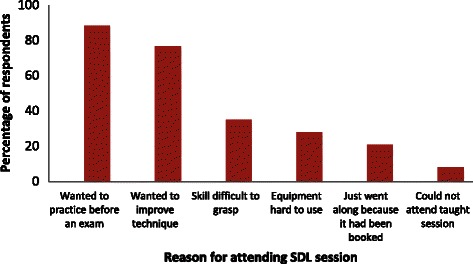
**Students’ (n = 86) reasons for attending SDL sessions for clinical skills.**

The three most commonly practised skills reported by respondents were eye, obstetric and ear examination, for which simulator equipment is available in the clinical skills lab. Types of skills never practised included hand washing and examination of joints. When asked to select factors that may have prompted them to attend an SDL session, desire to improve technique prior to assessment was selected by 88.4% followed by a wish to improve technique generally (76.7%) (Figure 3).

Indeed, 88.4% of the respondents agreed or strongly agreed that SDL sessions had improved their performance of the specific clinical skills being practised, with 79% agreed or strongly agreed that those sessions enhanced their mastery of clinical skills in general.

The dominant themes emerging from the free text comments related to the booking process (times and number of slots available particularly before examinations) and issues with equipment (the availability and ease of use of equipment). The benefits and limitations of clinical skills SDL also emerged as themes. The limitations included not having a tutor present for feedback and not being a good substitute for clinical practice on a real patient. The benefits included increasing confidence, practising in a relaxed atmosphere and accessing equipment to practise with.

## Discussion

There has been considerable discussion as to the applicability and efficacy of self-directed learning of clinical skills [21], allied to recognition of the challenges associated with students having the requisite self-awareness to take responsibility for determining their own learning needs and, indeed, the readiness of students to assume that role [34]. In this study, the participants were exclusively graduate entry medical students but from diverse primary degree disciplines and with varying levels of post-graduate experience. Despite variations in the composition of student classes beginning medical studies in 2008, 2009 and 2010, and the assumed attitudinal differences towards didactic and self-directed learning that such variation may bring, a year on year decline in engagement with clinical skills SDL was noted (Table 1). This could be interpreted as being reflective of disparity between simulation in medical teaching and practice in a supervised clinical setting, as reported elsewhere [18], which also emerged as a theme from the free text comments of students. This decline in engagement may also be as a result of increasing student numbers in each cohort and reduced access to the SDL labs, another theme that emerged from students’ free text comments.

Survey findings in relation to the types of skills most practised as reported by students mirrored our observation from analyzing SDL bookings that the most requested skills in SDL are those that require equipment or access to simulators. In our survey, the greatest stated impetus for engagement in clinical skills SDL was the desire to improve technique prior to assessment (selected as a factor by 88.4% of respondents), and the majority of respondents (88.4%) agreed that SDL sessions had improved their performance of the specific skill being practised.

However, our analysis did not indicate any statistically significant effect of SDL engagement on OSCE performance (Tables 2 and 3) for most of the clinical skills examined. There is, therefore, a possibility that students may be compounding errors/uncertainties while practising skills unsupervised. Alternatively, it is possible that those students who may be academically weaker are accessing SDL to work on improving skills they feel they are weaker in, and hence any improvement they achieve may just bring their proficiency up to the mean of the group. A limitation of this analysis is that it only included one examination year (2010/11) and that the students who engaged or not with SDL were not compared across other characteristics e.g. prior learning experiences or previous examination performances. Our finding that BLS results were lower (although not significantly) for second year students mirrors the deterioration in retention of key BLS skills amongst pre-clinical medical students over time observed elsewhere [35].

There is an argument for the development of strategies to promote greater engagement with clinical skills SDL, even if solely to avoid the financial implications of requiring additional clinical tutors for increased formal clinical skills training. In light of the results of this study, possibilities such as supplementary workshops [36], allied to encouragement of learning portfolio use by students such that they reflect on their progress, diagnose learning needs and create learning plans [10], could potentially result in an overall increased use of SDL . That said, records of the timing of personal SDL bookings indicated that imminent clinical skills examinations incentivised engagement with SDL considerably, particularly in the second pre-clinical year. As this is the third time that students will have participated in OSCEs, the observed enhanced interest in SDL may be due to increased awareness of skill deficits and plans to mitigate these [37] or reflect assumption of greater responsibility for their own learning over time [38]. Introducing more informal/formative testing throughout the academic year may be another option to increase SDL engagement among students.

The limitations of this study include a low response rate to the cross-sectional survey (possibly reflecting fatigue associated with over-survey of students in a newly established medical school). Also the booking of equipment for an SDL session, or indeed attending a SDL session, does not necessarily mean the students actively engaged with the clinical skill during the session, and therefore, it cannot be concluded that those students who booked or simply attended an SDL session demonstrated a greater degree of self-directed learning. As it currently stands, students are not required to submit logs of their SDL activity, and there is no other system in place to record actual use of equipment. A further limitation is that students’ motivation to attend may simply reflect their wish to improve performance in a skill they feel they are weak at, or to prepare for an imminent examination, rather than reflect a greater degree of self-directedness.

## Conclusions

Self-directed learning in clinical skills can provide an opportunity to build confidence and to improve techniques. While imminent clinical skills examinations appear to incentivise engagement with SDL, this engagement does not appear to improve exam performance for many clinical skills. More research is needed to determine which types of skills are best suited to using an SDL approach.
